# Nutrition and Physical Activity in the University Population: A Scoping Review of Combined Impacts on Psychological Well-Being, Cognitive Performance, and Quality of Life

**DOI:** 10.3390/jfmk10040374

**Published:** 2025-09-27

**Authors:** Paride Vasco, Salvatore Allocca, Claudia Casella, Francesco Paolo Colecchia, Maria Ruberto, Nicola Mancini, Maria Casillo, Antonietta Messina, Marcellino Monda, Giovanni Messina, Vincenzo Monda, Antonietta Monda, Fiorenzo Moscatelli, Rita Polito

**Affiliations:** 1 Department of Humanities, University of Foggia, 71100 Foggia, Italy; paride.vasco@unifg.it; 2 Department of Experimental Medicine, Section of Human Physiology and Unit of Dietetics and Sports Medicine, University of Campania “Luigi Vanvitelli”, 80131 Naples, Italy; salvatore.allocca1@unicampania.it (S.A.); francescopaolocolecchia@gmail.com (F.P.C.); maria.casillo@unicampania.it (M.C.); marcellino.monda@unicampania.it (M.M.); giovanni.messina@unicampania.it (G.M.); 3 Department of Advanced Biomedical Sciences, University of Naples “Federico II”, 80131 Naples, Italy; claudia.casella@unina.it; 4 Department of Education and Sport Sciences, Pegaso Telematic University, 80143 Naples, Italy; maria.ruberto@unipegaso.it (M.R.); nicola.mancini@unipegaso.it (N.M.); 5 Department of Precision Medicine, University of Campania “Luigi Vanvitelli”, 80138 Naples, Italy; antonietta.messina@unicampania.it; 6 Department of Economics, Law, Cybersecurity, and Sports Sciences, University of Naples “Parthenope”, 80133 Naples, Italy; vincenzo.monda@uniparthenope.it; 7 Department of Human Science and Quality of Life Promotion, San Raffaele Telematic University, 00166 Rome, Italy; antonietta.monda@uniroma5.it; 8 Department of Psychology and Health Sciences, Pegaso Telematic University, 80143 Naples, Italy; rita.polito@unipegaso.it

**Keywords:** nutrition, eating habits, mental health, quality of life, physical activity

## Abstract

**Background:** University students are particularly vulnerable to psychological distress due to the transitional nature of this life phase and increasing academic, social, and financial pressures. Accumulating evidence indicates that lifestyle behaviors—especially nutrition and physical activity—play a critical role in shaping mental health, cognitive functioning, and overall well-being in this population. **Methods:** The objective of this scoping review was to systematically map the literature on the combined impacts of diet and physical activity on psychological well-being among university students. Following PRISMA-ScR guidelines, an initial search of three major databases (PubMed, Sciencedirect, and Wiley) yielded 718 articles. After a multi-stage screening process, 39 articles of various designs (including cross-sectional, interventional, and review studies) focusing on non-clinical student populations were included. The studies were then thematically analyzed. **Results:** While most research explored isolated behaviors, a smaller set of integrated studies revealed synergistic effects, reporting enhanced outcomes in mental health and quality of life. Notably, several articles proposed practical strategies—such as app-based tools, structured wellness initiatives, and interdisciplinary educational programs—as effective means to support healthier habits. **Conclusions:** The evidence strongly suggests that universities should prioritize holistic, multi-component wellness strategies over siloed, single-behavior initiatives. Developing integrated programs that combine nutritional education and physical activity support represents a practical and effective approach to enhance student well-being.

## 1. Introduction

University is a critical transition period from adolescence to young adulthood, characterized by significant changes and challenges [1]. Students face increasing independence, new academic responsibilities, social pressures and, in many cases, their first experience of living away from home [2]. This rapidly changing environment exposes individuals to numerous stressors, including heavy course loads, performance expectations, financial difficulties, and the need to build new social networks [3]. These pressures can increase vulnerability to the development of mental health problems, making this population a crucial target for health promotion interventions. The scientific literature has extensively documented how this stage of life is associated with an increase in anxiety, depression and perceived stress approximately one-third of university students experience clinically significant mental health symptoms [4]. In response to new challenges, many university students adopt lifestyles that can compromise their long-term health. Eating habits tend to deteriorate, with a reduction in fruit and vegetable consumption and an increase in the intake of ultra-processed foods, rich in sugar, fat and salt [5]. Simultaneously, this period is often marked by a significant decline in physical activity and a rise in sedentary behaviors, driven by academic demands and increased screen time. Factors such as lack of time, economic constraints, poor cooking skills and easy access to ready-made, low-cost foods contribute to this deterioration in diet quality [6].

### 1.1. Impact on Mental Health, Cognitive Function and Quality of Life

Nutrition and physical activity are recognized as key determinants not only of physical health, but also of psychological well-being [7]. A growing body of research indicates that poor diet and sedentary lifestyles are associated with a higher prevalence of depressive symptoms, anxiety and stress among students. Conversely, a balanced diet, such as the Mediterranean diet, and regular physical activity are linked to improved mood, greater psychological resilience and better sleep quality, the latter being closely related to mental health [8]. In addition to psychological well-being, a healthy lifestyle can positively influence cognitive performance. Although research in this field is still developing, some evidence suggests that proper nutrition and exercise can improve executive functions such as attention, memory, and concentration, which are essential factors for academic success. Consequently, students’ perceived quality of life is intrinsically linked to their daily habits [9].

### 1.2. Purpose of the Review

While numerous reviews have investigated the isolated effects of either diet or physical activity, a critical gap persists in the scientific literature: the combined and synergistic impacts of these behaviors on university students’ health remain largely unexplored. Most existing research syntheses and interventions continue to treat these interdependent lifestyles as separate entities, thus overlooking their complex interactions, which may produce effects greater than the sum of their individual parts [10,11]. Therefore, the primary contribution of this scoping review is to address this specific gap. We aim to systematically map the existing literature on the combined impacts of nutrition and physical activity on psychological well-being, cognitive performance, and quality of life in the university population. Our goal is to provide a clear summary of the current, often fragmented, knowledge, explicitly identify the under-researched areas, and build a solid foundation for the development of integrated and effective health interventions within universities.

## 2. Materials and Methods

**Figure 1:**
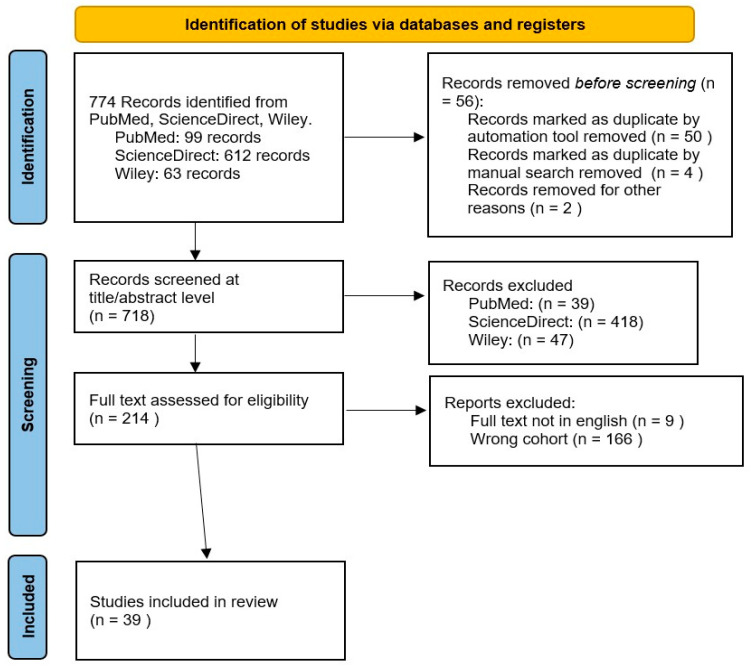
PRISMA flow chart of the conducted search. The figure illustrates a PRISMA flow diagram documenting the systematic review process, including the identification, screening, and inclusion stages.

This scoping review was conducted following the PRISMA (Preferred Reporting Items for Systematic reviews and Meta-Analyses) method [12]. A literature search was performed between June and July 2025 across the Sciencedirect, PubMed, and Wiley databases (this combination was chosen to ensure a comprehensive and multidisciplinary coverage, leveraging the breadth of PubMed alongside the extensive collections of major publishers), using the following search algorithm: (“nutrition” OR “diet” OR “eating habits”) AND “university students” AND (“physical activity” OR “exercise”) AND “mental health” AND (“quality of life” OR “QoL”). The literature search was performed by one author and independently verified by a second author. As shown in the PRISMA flowchart 1, the initial search yielded 612 articles from Sciencedirect (meeting the inclusion criteria: published after 2010; review/article type; written in English; and available in open access), 99 from PubMed, and 63 from Wiley. After the removal of duplicates, the total number of unique articles was 718. These articles underwent a two-stage screening process. First, based on title and abstract screening, 214 articles were deemed relevant, including experimental/quasi-experimental, observational, and review studies. Subsequently, a full-text review of these 214 articles resulted in the exclusion of 175 papers due to an incorrect study population or the full text not being available in English. This process led to a final selection of 39 articles for inclusion in this scoping review (Figure 1).

### 2.1. Identification Phase

In this phase, a search was conducted according to the methods described, which yielded 774 records. After removing duplicates, 718 unique records were carried forward to the screening phase. This process was managed using the Rayyan software (https://www.rayyan.ai/, accessed on 10 August 2025, Rayyan Systems Inc., Cambridge, MA, USA), where three reviewers independently and blindly analyzed the records. Title/abstract screening and full-text assessment were performed independently and in blind by three reviewers using Rayyan software (Rayyan Systems Inc., Cambridge, MA, USA); disagreements were resolved by majority vote after discussion.

### 2.2. Screening Phase

During the screening stage, the 718 unique records were assessed for inclusion based on their titles and abstracts. The selection was guided by several criteria. Regarding the research area, only papers relevant to the purpose of this review were included, regardless of the specific disciplinary field (e.g., psychology, health, medicine). All study designs were considered, including experimental, quasi-experimental, observational, and review articles. The target population was required to consist of university students or have a specific focus on them. Furthermore, eligible articles had to investigate variables related to the review’s scope, such as quality of life, mental health, physical activity, and eating habits. Pertaining to publication details, inclusion was limited to full scientific journal articles published after 2010, written in English, and available as open-access full text. Essays, conference proceedings, posters, and abstracts were excluded. This screening process resulted in the selection of 214 articles for full-text eligibility assessment.

### 2.3. Inclusion Phase

The 214 articles that passed the initial screening were then subjected to a full-text review to determine final eligibility. At this stage, specific exclusion criteria were applied. Studies were excluded if they focused on the impacts of the COVID-19 pandemic, to avoid the influence of a unique and global confounding factor. Furthermore, to maintain a focus on prevention and health promotion in the general student population rather than clinical treatment, studies whose samples consisted exclusively of clinical populations with diagnosed mental disorders (e.g., Major Depressive Disorder, post-traumatic stress disorder) or other pathological conditions were also excluded. Therefore, for final inclusion, studies were required to examine the relationship between lifestyle factors (such as physical activity, exercise, and eating habits) and health outcomes (such as mental well-being, stress, and quality of life) within a non-clinical university student population. No restrictions were placed on the specific type of diet or physical activity intervention, as long as the study’s goal was to assess an effect on students’ well-being. This final eligibility assessment resulted in the exclusion of 175 articles, leading to a total of 39 studies included in this scoping review.

## 3. Results

A total of 39 studies were included in this scoping review, exploring the associations and combined impacts of nutrition and physical activity on psychological well-being, cognitive performance, and quality of life among university students. The included studies vary in design, population, and focus, yet can be grouped into three major thematic categories:

- **Separate Effects of Diet and Physical Activity:** Several studies explored the individual contributions of either dietary habits or physical activity. For example, Akter and Hossain (2024) [5] examined how dietary patterns correlate with sedentary behavior, while Al Ali and Khazaaleh (2023) [6] investigated food intake and lifestyle risks.
- **Psychological and Cognitive Outcomes:** Multiple studies assessed mental health outcomes such as stress, anxiety, and cognitive function. Ansari et al. (2025) [1] conducted a systematic review linking stress levels to emotional well-being, and Alexatou et al. (2025) [13] reviewed the psychological role of emotional eating.
- **Combined Interventions and Holistic Approaches:** A subset of studies focused on integrated approaches combining both diet and exercise. While fewer in number, these studies (e.g., Remskar et al., 2024 [7]) suggested that combined lifestyle modifications may have synergistic effects on mental health, resilience, and perceived quality of life.

To provide a clearer and more detailed overview of the evidence base, the included studies are summarized in Table 1, which classifies each article by its country or region of focus, and primary objective. This classification permits a richer analysis of the existing literature, highlighting the prevalence of cross-sectional designs and identifying geographical areas where research is concentrated or lacking.

Table 2 provides an overview of the studies included in this review, grouped by thematic category.

## 4. Discussion

This scoping review was designed to address a specific and significant gap in the literature: the lack of a comprehensive overview of the combined effects of nutrition and physical activity on the psychological well-being of university students. While previous research has extensively documented the individual effects of these behaviors, our findings confirm that their potential synergistic impact remains a critically under-represented area of investigation. A total of 39 articles, reported in the Table A1 of the Appendix A, were analyzed and grouped thematically based on their focus. The main contribution of this work, therefore, is not merely to reiterate known associations but to provide a clear map that exposes this research gap. By doing so, we underscore the urgent need for a paradigm shift—from fragmented, behavior-specific interventions to more holistic research models and public health strategies that target multiple behaviors simultaneously. The following discussion highlights key findings, connects them with broader research contexts, identifies gaps, and proposes directions for future investigations.

### 4.1. Overview of Key Findings

This review set out to address the limited understanding of how nutrition and physical activity jointly impact the psychological and cognitive health of university students. While previous research has extensively examined the individual effects of these behaviors, the current review sought to explore their potential synergistic outcomes—where their combined influence on mental well-being and quality of life may exceed the sum of their separate parts. Among the 39 studies included, a majority focused on the effects of either nutrition or physical activity independently. These works consistently showed that inadequate dietary patterns—such as low intake of fruits and vegetables and high consumption of processed foods—were associated with increased symptoms of depression, stress, and anxiety [8,17,23,29]. Similarly, low levels of physical activity and high sedentary behavior were frequently linked with poorer mental health outcomes and lower cognitive performance [3,4,39]. A substantial portion of studies also investigated psychological and cognitive consequences related to lifestyle habits. Emotional eating, perceived stress, and sleep disturbances emerged as recurring mediators between behavior and well-being. These findings reinforce the vulnerability of university students to mental health challenges during a life phase marked by academic, social, and financial pressures [13,27,40]. More notably, only a smaller segment of studies adopted an integrated approach that examined both nutrition and physical activity together. Despite their limited number, these studies provided strong support for the idea that combined lifestyle interventions—particularly those incorporating physical movement alongside dietary education or behavior change strategies—can generate significantly better outcomes in terms of mental health and quality of life.

The underlying reasons for this synergy are likely multifaceted, involving an intricate interplay of physiological and psychological mechanisms. Physiologically, physical activity enhances insulin sensitivity and glucose metabolism, which can stabilize mood and reduce the cravings for high-sugar, low-nutrient foods often associated with poor mental health. Simultaneously, a nutrient-dense diet provides the essential building blocks—such as omega-3 fatty acids, antioxidants, and B vitamins—that support neurotransmitter function and reduce systemic inflammation, a factor increasingly linked to depression. Exercise also promotes the release of endorphins and brain-derived neurotrophic factor (BDNF), which improve mood and cognitive function, respectively. A healthy diet, in turn, provides the metabolic fuel necessary to sustain physical activity and optimize post-exercise recovery. Psychologically, engaging in one positive health behavior can increase an individual’s self-efficacy and motivation to adopt another. For instance, the immediate mood boost from a workout (an acute effect of exercise) can empower a student to make healthier food choices later in the day. Conversely, feeling more energetic from an improved diet can lower the perceived barrier to engaging in physical activity. This creates a positive feedback loop where improvements in one domain reinforce and facilitate progress in the other, leading to more sustainable lifestyle changes and greater overall well-being than could be achieved by targeting either behavior in isolation.

This synergistic impact suggests the presence of reinforcing feedback loops between diet and exercise behaviors that deserve further exploration [11,18]. The scoping review thus confirms that while a fragmented body of literature exists on lifestyle and health in university settings, few studies have directly explored their interaction. The thematic categorization presented in Table 2 highlights this imbalance, illustrating the dominance of single-factor studies and the relative scarcity of research into combined behavioral impacts. These insights underscore the need for more integrated research designs and public health strategies targeting multiple behaviors simultaneously.

### 4.2. Interpretation in Light of Existing Literature

The findings align with previous research emphasizing the critical role of healthy behaviors in young adult populations. However, while several studies confirm the association between single lifestyle factors and psychological health, integrated models are still under-represented. This suggests that current interventions may be too narrowly focused, despite the well-documented interdependence of diet, exercise, and mental health. Furthermore, the variability in study designs and outcome measures indicates a need for standardized approaches in future investigations.

### 4.3. Practical Implications and Operational Recommendations

The findings of this review have important practical implications for designing health promotion strategies targeting university students. Evidence strongly supports the value of integrated approaches that combine nutritional education with physical activity programs. For instance, Remskar et al. (2024) [7] and Trottier et al. (2021) [11] demonstrated that interventions incorporating both exercise and dietary guidance—often enhanced with mindfulness or stress management components—can produce significantly greater improvements in mental health and overall quality of life than single-domain interventions. A number of studies provide examples of effective strategies already tested in university contexts. The BEST-U mobile health app developed by Forbush et al. (2023) [19], for example, was designed specifically for university students and showed promising results in promoting self-esteem and healthier eating behaviors through a user-centered approach. Similarly, the lifestyle redesign program described by Leigh An et al. (2024) [26] offered a structured method for supporting student well-being through behavioral change. On a systemic level, Martinez et al. (2022) [29] highlighted the positive impact of campus food pantries on students’ sleep, mental health, and physical well-being, suggesting that even logistical or infrastructural interventions can generate meaningful psychological benefits. Taken together, these findings point to a set of actionable recommendations. Universities should prioritize the implementation of multidisciplinary health programs that address both diet and physical activity in tandem, ideally supported by digital tools for monitoring and personalized feedback. Creating supportive environments—such as offering healthy food options on campus, facilitating access to physical activity facilities, and integrating health promotion into academic life—can further reinforce positive lifestyle choices. As the results of this review suggest, moving from fragmented, behavior-specific interventions to more holistic and student-centered strategies holds considerable promise for improving the mental and cognitive well-being of university students.

### 4.4. The Impact of Heterogeneity and Moderating Factors

The studies included in this review exhibit considerable heterogeneity in terms of research design, geographical location, and specific student populations. This variability, while enriching the scope of our findings, also presents challenges for synthesis and generalization. For instance, the cross-sectional studies, which form a substantial part of the included literature, can only establish associations, while the few interventional studies provide stronger evidence for causality [5,24]. The effectiveness of an intervention tested in a European context may not be directly transferable to student populations in Asia or the Middle East without significant cultural adaptation. Therefore, while the overall trend indicates a positive relationship between healthy lifestyles and well-being, the practical application of these findings must be context-specific [6]. Furthermore, our review suggests that the relationship between nutrition, physical activity, and student well-being is likely moderated by several factors that were not consistently addressed in the included literature. As the reviewer noted, variables such as the socioeconomic status of students (influencing access to healthy food and gyms), their field of study (e.g., health sciences versus humanities, which may correlate with different levels of health literacy), and the broader cultural context (shaping dietary norms and attitudes towards exercise) are critical. For example, a student facing financial hardship may prioritize low-cost, processed foods over healthier options, regardless of their knowledge. The under-exploration of these moderating factors represents a significant research gap, and future studies should aim to investigate these variables more systematically to develop more tailored and equitable health promotion strategies.

### 4.5. Research Gaps, Future Directions, and Limitations

This scoping review systematically mapped a broad and multidisciplinary body of literature on the relationship between nutrition, physical activity, and psychological and cognitive outcomes in university students. Through this process, several research gaps have emerged that warrant further investigation. To address these gaps effectively, future research must move beyond general recommendations and adopt more rigorous and specific designs. We propose the following prescriptive directions:

- Specific Study Designs: There is an urgent need for longitudinal cohort studies that track first-year students throughout their university careers to understand the temporal dynamics between variables. Furthermore, Randomized Controlled Trials (RCTs) are essential to establish causality.
- Concrete Research Questions: Future RCTs could address specific questions such as: ‘*Does a 12-week integrated intervention, combining nutritional education with group physical activity, lead to a greater reduction in anxiety symptoms compared to a single-component intervention?*’
- Particular Populations: Research should explicitly focus on underrepresented and potentially more vulnerable student populations, such as international students, students from low socioeconomic backgrounds, and those in non-health-related academic fields.

Notably, there is a lack of longitudinal and interventional studies specifically assessing the combined effects of diet and physical activity. Additionally, the literature shows an underrepresentation of diverse student populations, including variation by socioeconomic background, field of study, and cultural context. Another significant gap is the limited exploration of the mechanisms linking lifestyle behaviors to cognitive performance and academic success. To address these limitations, future research should adopt more integrative and multidisciplinary approaches capable of capturing the complexity of interactions between behaviors and health outcomes. Incorporating both behavioral and physiological data, as well as standardized measures, would enhance comparability across studies and deepen our understanding of causal relationships. While this review provides a comprehensive overview of existing evidence, it also has certain limitations. It did not include a formal quality assessment of the studies, limiting the strength of conclusions regarding evidence reliability. While this is a common feature of scoping reviews, a general critical appraisal of the included literature reveals important patterns that contextualize our findings. A significant portion of the included studies employed cross-sectional designs. This design is valuable for identifying associations but makes it impossible to establish causality; for example, we cannot determine whether poor nutrition leads to stress or if stress leads to poor nutritional choices. Furthermore, many studies were conducted on specific, often small and homogenous, student populations within a single institution, which may limit the generalizability of the findings to the broader, more diverse international university population. Therefore, while this review successfully maps the existing evidence, the reader should interpret these findings with caution. The predominance of observational studies underscores a critical gap—the need for more robust, longitudinal, and interventional research (e.g., randomized controlled trials) to establish causal links and test the efficacy of combined interventions in a controlled manner. Moreover, language restrictions and potential publication bias may have excluded relevant studies. Despite these constraints, the review offers a valuable foundation for the development of targeted and evidence-based health promotion strategies within university settings.

## 5. Conclusions and Practical Recommendations

In conclusion, this scoping review systematically mapped the literature on the impacts of nutrition and physical activity on university students’ well-being. Our analysis confirms that while the benefits of each behavior are well-documented, the scientific literature remains fragmented, with a predominant focus on single-factor studies. A smaller but significant subset of studies, however, provides evidence for synergistic effects, suggesting that integrated interventions yield superior outcomes for mental health and quality of life. The central implication of these findings is the need for a paradigm shift in how university wellness is approached—moving away from siloed, behavior-specific initiatives toward holistic, multi-component strategies that address lifestyle in an integrated manner. To translate these findings into concrete actions, we have developed a set of practical recommendations for key stakeholders within the university ecosystem. The following Table 3 outlines specific, evidence-based strategies for university administrators, campus health professionals, and students themselves, aiming to foster a healthier and more supportive academic environment. Ultimately, by adopting such integrated and evidence-based approaches, universities can play a pivotal role in promoting lifelong health and well-being for the next generation of leaders and professionals. Furthermore, the geographical mapping of the included studies, presented in Table 1, reveals a concentration of research in North America, Europe, and Asia, with a notable lack of evidence from other regions such as Africa and South America. This highlights the need for future research to be more globally representative to ensure that health promotion strategies are culturally relevant and effective for diverse student populations worldwide.

## Figures and Tables

**Table 1:** Summary of Included Studies by Design, Country/Region, and Primary Focus.

First Author (Year)	Country/Region	Primary Focus
AboElela (2025) [14]	Saudi Arabia	Migraine prevalence & academic impact
Akter (2024) [5]	Bangladesh	Dietary patterns & sedentary behavior
Al Ali (2023) [6]	Jordan	Food consumption & health-risk behaviors
Alexatou (2025) [13]	N/A	Emotional eating among students
Ansari (2025) [1]	N/A	Stress & emotional well-being
Brinsley (2025) [3]	N/A	Digital lifestyle interventions for mental health
Dakanalis (2025) [8]	Greece	Mediterranean diet, stress & sleep quality
deJonge (2020) [15]	Canada	Perceptions of physical activity for mental health
Di Santi (2025) [16]	Brazil	Longitudinal evaluation of mental well-being
El Mikkawi (2024) [17]	Lebanon	Mediterranean diet & mental health
Estrela (2025) [18]	N/A	Educational interventions & health literacy
Forbush (2023) [19]	USA	mHealth app for eating disorders
Harris (2024) [20]	N/A	Well-being interventions for pharmacy students
Hsin (2020) [21]	Taiwan	Students’ conceptions of health
Huang (2024) [22]	China	Body appreciation & intuitive eating
Isaacs (2025) [23]	USA	Plant-based diet intervention
Jeftic (2023) [24]	Australia	Exercise service for students with mental distress
Lacerda (2025) [25]	Brazil	Students’ perceptions of yoga on mental health
Leigh An (2024) [26]	Korea	Lifestyle redesign program for well-being
Li (2024) [27]	China	Sleep, breakfast skipping & depressive symptoms
López-Moreno (2021) [28]	Spain	Eating habits, alcohol & academic performance
Martinez (2022) [29]	USA	Campus food pantry use & health outcomes
Navarra-Ventura (2024) [2]	Spain	Factors for high and low mental well-being
Nazari (2024) [30]	N/A	Laughter yoga for stress & anxiety
O’Hara (2021) [31]	Qatar	Health at Every Size^®^ informed activity
Pinto (2021) [32]	N/A	Perceptions of food healthiness
Radhamani (2020) [33]	India	Neural dynamics of mind–body relaxation
Rahimi (2024) [4]	Afghanistan	Lifestyle factors & mental health
Remskar (2024) [7]	N/A	Combined physical activity & mindfulness interventions
Sifat (2022) [34]	Bangladesh	Digital health tools for mental health
Stonerock (2024) [35]	N/A	Exercise as therapy for anxiety
Tan (2020) [10]	Germany	Physical activity, sedentary time & perceived stress
Teodoro (2021) [36]	N/A	Grazing behaviors & psychopathology
Trottier (2021) [11]	Canada	Web-based wellness platform for first-year students
Verma (2022) [37]	India	Physical activity patterns & curriculum perceptions
Wang (2013) [38]	China	Healthy lifestyles & influential factors
Ye (2023) [39]	USA	Exercise & psychotic symptoms
Zhang (2023) [40]	China	Circadian rhythms, sleep quality & lifestyle
Zhou (2020) [9]	USA	Development of a university well-being scale

Note: A summary of the 39 studies included in this scoping review. Each study is classified according to its country or region, and primary focus. N/A (Not Applicable) is used for systematic reviews or meta-analyses that do not have a specific geographical focus. The ‘Primary Focus’ column offers a concise summary of the main objective detailed in Appendix A.

**Table 2:** Categorization of included articles by thematic focus.

Category	Authors (Year)
Combined interventions and holistic approaches	Di Santi et al. (2025) [16]; Estrela et al. (2025) [18]; Forbush et al. (2023) [19]; Harris et al. (2024) [20]; Jeftic et al. (2023) [24]; Leigh An et al. (2024) [26]; Radhamani et al. (2020) [33]; Remskar et al. (2024) [7]; Sifat et al. (2022) [34]; Stonerock et al. (2024) [35]; Tan et al. (2020) [10]; Trottier et al. (2021) [11]
Psychological and Cognitive outcomes	AboElela et al. (2025) [14]; Alexatou et al. (2025) [13]; Ansari et al. (2025) [1]; Brinsley et al. (2025) [3]; Hsin et al. (2020) [21]; Huang et al. (2024) [22]; Lacerda et al. (2025) [25]; Li et al. (2024) [27]; Navarra-Ventura et al. (2024) [2]; Nazari et al. (2024) [30]; O’Hara et al. (2021) [31]; Rahimi et al. (2024) [4]; Teodoro et al. (2021) [36]; Ye et al. (2023) [39]; Zhou et al. (2020) [9]; deJonge et al. (2020) [15]
Separate effects of diet and physical activity	Akter et al. (2024) [5]; Al Ali et al. (2023) [6]; Dakanalis et al. (2025) [8]; El Mikkawi et al. (2024) [17]; Isaacs et al. (2025) [23]; López-Moreno et al. (2021) [28]; Martinez et al. (2022) [29]; Pinto et al. (2021) [32]; Verma et al. (2022) [37]; Wang et al. (2013) [38]; Zhang et al. (2023) [40]

Note: Articles are grouped based on their primary thematic focus as determined by study objectives and methodology. Some studies may conceptually overlap multiple categories but are listed under their dominant theme for clarity.

**Table 3:** Actionable Recommendations for University Stakeholders.

Stakeholder	Recommendation	Rationale & Supporting Evidence
University Administration	Integrate Health & Wellness into Curriculum: Implement mandatory for-credit wellness modules for first-year students covering nutrition, physical activity, and stress management. Improve Campus Food Environment: Subsidize healthy food options in cafeterias and ensure the availability of campus food pantries to combat food insecurity.	Fosters a proactive culture of health from the start of the university journey. Educational interventions can improve health literacy and lifestyle choices [18]. Infrastructural interventions like food pantries have been shown to improve students’ sleep, mental health, and physical well-being [29].
Campus Health Professionals	Establish Peer-Led Wellness Groups: Create student-led clubs focused on healthy cooking, group fitness (e.g., running or yoga clubs), or mental health advocacy. Leverage Digital Health Tools: Promote and provide access to evidence-based mobile health apps for tracking behaviors, providing personalized feedback, and offering support.	Peer support is a powerful motivator for behavioral change and can create a supportive campus community, reducing feelings of isolation [21,24]. App-based tools are a scalable and effective way to promote healthier habits and self-esteem among students in a user-centered manner [19].
Students & Student Organizations	Establish Peer-Led Wellness Groups: Create student-led clubs focused on healthy cooking, group fitness (e.g., running or yoga clubs), or mental health advocacy. Advocate for Systemic Change: Use student government and advocacy groups to lobby for better access to mental health services, improved gym facilities, and healthier food options on campus.	Peer support is a powerful motivator for behavioral change and can create a supportive campus community, reducing feelings of isolation [7]. Empowers students to take an active role in shaping a healthier university environment for themselves and their peers [11].

## Data Availability

Not applicable.

## Appendix A

**Table A1:** Full list of included articles with objectives.

Title	Authors	Year	Objective
Migraine among King Khaled University students; prevalence, determinants, and impact on academic life	AboElela, A.M.; Mohamed, S.S.; Alsaleem, S.A.; Aboareef, R.A.M.; Al Hunaif, G.M.; Alshehri, Y.A.S.; Almazni, T.A.A.; Alshmrani, L.S.; Alqahtani, R.M.; Alshehri, L.M.A.; Dashnan, L.D.; Alshehri, S.A.S.; Al manea, D.M.; Alahmari, G.S.; Ghazy, R.M.	2025	Determine how prevalent migraine is among students at King Khalid University, identify its contributing factors, and evaluate the impact of migraine and other types of headache on academic performance and university life.
Food Consumption Patterns and Sedentary Behaviors Among the University Students: A Cross-Sectional Study.	Akter, M.M.; Hossain, M.J.	2024	Explore dietary habits and sedentary behavior among university students in Dhaka, Bangladesh.
Assessment of food consumption, smoking, alcohol use, and physical activity by sex and major of study among a sample of college students in Jordan	Al Ali, N.M.; Khazaaleh, F.K.	2023	Examine how gender and field of study (health-related vs. non-health) influence the frequency of food intake and engagement in health-risk behaviors among undergraduate students.
Exploring the Impact of Emotional Eating Among University Students: A Literature Review	Alexatou, O.; Papadopoulou, S.K.; Mentzelou, M.; Deligiannidou, G.-E.; Dakanalis, A.; Giaginis, C.	2025	Critically evaluate existing clinical evidence on how emotional eating affects university students.
Association of stress and emotional well-being in non medical college students: a systematic review and meta-analysis	Ansari, S.; Khan, I.; Iqbal, N.;	2025	Examine the relationship between stress and emotional well-being in non-medical college students, while exploring variability among studies through subgroup analyses and meta-regression techniques.
Effectiveness of Digital Lifestyle Interventions on Depression, Anxiety, Stress, and Well-Being: Systematic Review and Meta-Analysis	Brinsley, J.; O’Connor, E.J.; Singh, B.; McKeon, G.; Curtis, R.; Ferguson, T.; Gosse, G.; Willems, I.; Marent, P.J.; Szeto, K.; Firth, J.; Maher, C.	2025	Valuate how digital lifestyle interventions influence depression, anxiety, stress, and overall well-being in adults, and identify key technological, methodological, and demographic factors linked to variations in mental health outcomes.
Mediterranean Diet Compliance Is Related with Lower Prevalence of Perceived Stress and Poor Sleep Quality in University Students: A Cross-Sectional Study in Greece	Dakanalis, A.; Papadimitriou, K.; Alexatou, O.; Deligiannidou, G.-E.; Pappa, M.; Papadopoulou, S.K.; Louka, A.; Paschodimas, G.; Mentzelou, M.; Giaginis, C.	2025	Examine how adherence to the Mediterranean diet relates to perceived stress and sleep quality in Greek university students, while also analyzing its associations with different sociodemographic and lifestyle variables.
University students’ and clinicians’ beliefs and attitudes towards physical activity for mental health	deJonge, M.L.; Omran, J.; Faulkner, G.E.; Sabiston, C.M.	2020	Investigate how university students and mental health professionals perceive and value physical activity as a tool for supporting mental health.
Measuring Mental Health in 2 Brazilian University Centers: Protocol for a Cohort Survey	Di Santi, T.; Nascimento, A.G.; Fukuti, P.; Marchisio, V.; Araujo do Amaral, G.C.; Peternella Vaz, C.F.; Finotti Carrijo, L.D.; de Oliveira, L.C.; da Costa, L.O.; Mancini Marion Konieczniak, E.; Zuppi Garcia, L.A.; Cabrelon Jusevicius, V.C.; de Castro Humes, E.; Rossi Menezes, P.; Miguel, E.; Caye, A.	2025	Outline the methodology of a web-based longitudinal survey aimed at evaluating mental well-being, tracking the occurrence of psychological symptoms, and identifying related factors within a sample of Brazilian university students.
Adherence to the Mediterranean diet and mental health among university students in Lebanon	El Mikkawi, H.; El Khoury, C.; Rizk, R.	2024	Examine how adherence to the Mediterranean diet relates to mental health indicators—namely depression, anxiety, and stress—among university students in Lebanon.
Educational interventions for the adoption of healthy lifestyles and improvement of health literacy: a systematic review	Estrela, M.; Leitão, C.; Neto, V.; Martins, B.; Santos, J.; Branquinho, A.; Figueiras, A.; Roque, F.; Herdeiro, M.T.	2025	Evaluate how educational interventions influence health literacy and the adoption of healthier lifestyle choices, while also examining their impact on attitudes, behaviors, and overall health outcomes.
The Building Healthy Eating and Self-Esteem Together for University Students Mobile App to Treat Eating Disorders: User-Centered Research Design and Feasibility Study	Forbush, K.T.; Christensen Pacella, K.A.; Thomeczek, M.L.; Gould, S.R.; Chapa, D.A.N.; Richson, B.N.; Perko, V.L.; Ayres, J.; Chen, Y.; Negi, S.	2023	Detail the creation, functionality, and user acceptance of the BEST-U mobile health (mHealth) application, developed to bridge the gap in eating disorder treatment availability within university settings.
Scoping Review of Programmatic Well-Being Interventions and Outcomes to Support Pharmacy Students	Harris, S.C.; Gandavarapu, S.; Zeeman, J.M.	2024	Examine how various strategies adopted by pharmacy schools contribute to enhancing the well-being of their students.
Students’ conceptions of health: A cross educational stage survey	Hsin, M.; Lin, C.; Li, H.; Lin, S.	2020	Examine students’ understanding of health across junior high, senior high, and college levels in Taiwan using a cross-sectional survey, with particular attention to four key dimensions: physical, mental, social, and cultural well-being.
The gratitude model of body appreciation and intuitive eating: Replication and extension of the model to explain intuitive eating facets among young adult women in China	Huang, Z.; Wang, S.; Lin, Y.; Cui, T.; Barnhart, W.R.; Gaggiano, C.M.; Ji, F.; He, J.	2024	Replicate and expand the gratitude-based model linking body appreciation to intuitive eating among Chinese women, and investigate its capacity to account for the different dimensions of intuitive eating behavior.
Development and Implementation of a 3-Week Whole-Food Plant-Based Vegan Diet Intervention for College Students	Isaacs, S.E.; Bogardus, M.; Thompson, M.; Wu, S.; Howell, M.	2025	“Detail the methodology of the Diet and Health Study, a pilot feasibility project designed to evaluate how a whole-food, plant-based vegan diet influences college students’ physical and mental well-being. The study also aimed to assess both the practicality of its implementation and participants’ acceptance of the intervention, while comparing potential differences in outcomes based on two distinct modes of educational delivery.
The Stride program: Feasibility and pre-to-post program change of an exercise service for university students experiencing mental distress	Jeftic, I.; Furzer, B.; Dimmock, J.A.; Wright, K.; Budden, T.; Boyd, C.; Simpson, A.; Rosenberg, M.; Sabiston, C.M.; de Jonge, M.; Jackson, B.	2023	Evaluate the practicality of introducing an on-campus exercise initiative tailored for students experiencing mental distress, and examine pre- to post-program variations in mental and physical health, as well as other related outcomes.
Experiences and perceptions of college students regarding the effects of Shivam Yoga on mental health and behavior: A qualitative study	Lacerda, K.C.D.; de Assis, A.D.; Souza, G.G.L.	2025	Explore university students’ perceptions of how yoga influences their physical well-being, emotional states, and everyday life, with the aim of gaining insight into their lived experiences.
A Quasi-Experimental Study Investigating the Impact of a Lifestyle Redesign Program on the Well-Being of Korean University Students	Leigh An, S.; Kim, G.;	2024	Evaluate how a lifestyle redesign intervention affects the overall well-being of university students in Korea.
Associations between insufficient sleep, skipping breakfast and depressive symptoms in children and adolescents: A school-based cross-sectional study in China	Li, S.; Li, X.; Wang, J.; Jiang, D.; Zhang, Y.; Lou, W.; Bao, K.; Gong, Q.	2024	Explore both the individual and combined relationships between inadequate sleep and breakfast skipping and their link to depressive symptoms, while also assessing potential variations based on age and gender.
Influence of eating habits and alcohol consumption on the academic performance among a university population in the community of Madrid: A pilot study	López-Moreno, M.; Garcés-Rimón, M.; Miguel, M.; Iglesias-López, M.T.	2021	Analyze how students’ eating behaviors and alcohol intake correlate with their academic performance within a university population.
Campus Food Pantry Use Is Linked to Better Health Among Public University Students	Martinez, S.M.; Chodur, G.M.; Esaryk, E.E.; Kaladijian, S.; Ritchie, L.D.; Grandner, M.	2022	Investigate, through retrospective analysis, the potential relationship between the use of a campus food pantry and enhancements in sleep quality, mental well-being, and overall physical health among students attending a public university.
Factors associated with high and low mental well-being in Spanish university students	Navarra-Ventura, G.; Riera-Serra, P.; Roca, M.; Gili, M.; García-Toro, M.; Vilagut, G.; Alayo, I.; Ballester, L.; Blasco, M.J.; Castellví, P.; Colom, J.; Casajuana, C.; Gabilondo, A.; Lagares, C.; Almenara, J.; Miranda-Mendizabal, A.; Mortier, P.; Piqueras, J.A.; Soto-Sanz, V.; Alonso, J.	2024	Investigate the determinants linked to both elevated and diminished levels of mental well-being within a representative group of first-year university students in Spain
The effect of laughter yoga on stress and anxiety of nursing students: A systematic review	Nazari, A.M.; Ghazanfari, M.J.; Zeydi, A.E.; Zare-Kaseb, A.	2024	Assess how laughter yoga influences stress and anxiety reduction among nursing students.
Evaluating the impact of a brief Health at Every Size^®^-informed health promotion activity on body positivity and internalized weight-based oppression	O’Hara, L.; Ahmed, H.; Elashie, S.	2021	Evaluate the impact of brief health promotion activity informed by the Health at Every Size^®^ approach on body positivity and internalized weight-based oppression among Qatar University female students.
Perceived healthiness of foods: A systematic review of qualitative studies	Pinto, V.R.A.; Campos, R.F.A.; Rocha, F.; Emmendoerfer, M.L.; Vidigal, M.C.T.R.; da Rocha, S.J.S.S.; Della Lucia, S.M.; Cabral, L.F.M.; de Carvalho, A.F.; Perrone, Í.T.	2021	Analyze how perceptions of food healthiness have been studied, examine their influence on consumer attitudes and dietary choices, and understand their role in shaping consumer expectations.
Computational analysis of cortical EEG biosignals and neural dynamics underlying an integrated mind–body relaxation technique	Radhamani, R.; Nizar, N.; Kumar, D.; Pillai, G.S.; Prasad, L.S.; Jitha, S.S.; Kuniyil, M.K.V.; Sekhara, A.A.; Kumar, V.S.; Pillai, S.; Diwakara, S.	2020	Examine brain activity patterns during a brief integrated yoga and meditation session to evaluate dynamic neural changes and how these practices influence brain organization in healthy individuals. Additionally, the study aimed to investigate the spatial and temporal effects of a Kerala-origin yoga and meditation method.
Assessing the relationship between lifestyle factors and mental health outcomes among Afghan university students	Rahimi, A.; Wardak, M.F.; Shayan, N.A.	2024	Investigate the relationship between key lifestyle behaviors—such as physical activity, dietary patterns, and sleep—and mental health indicators including depression, anxiety, and stress among university students at Herat University in Afghanistan. The study posited that poorer health behaviors and demographic factors would be linked to elevated psychological distress.
Effects of combining physical activity with mindfulness on mental health and wellbeing: Systematic review of complex interventions	Remskar, M.; Western, M.J.; Osborne, E.L.; Maynard, O.M.; Ainsworth, B.	2024	Critically assess existing evidence on the effects of interventions integrating physical activity and mindfulness on mental health and well-being, with an additional focus on uncovering the mechanisms through which these combined approaches exert their influence.
Motivations Toward Using Digital Health and Exploring the Possibility of Using Digital Health for Mental Health in Bangladesh University Students: Cross-sectional Questionnaire Study	Sifat, M.S.; Saperstein, S.L.; Tasnim, N.; Green, K.M.	2022	Explore the acceptability of digital health tools for mental health promotion among university students in Bangladesh, and examine the underlying motivational factors influencing their use.
Is exercise a viable therapy for anxiety? Systematic review of recent literature and critical analysis	Stonerock, G.L.; Gupta, R.P.; Blumenthal, J.A.	2024	Critically re-evaluate the role of exercise in managing anxiety through a systematic review of randomized controlled trials.
Independent and Combined Associations of Physical Activity, Sedentary Time, and Activity Intensities With Perceived Stress Among University Students: Internet-Based Cross-Sectional Study	Tan, S.L.; Jetzke, M.; Vergeld, V.; Müller, C.	2020	Investigate the relationships between physical activity, sedentary behavior, and perceived stress levels among university students in Germany, while accounting for sociodemographic and lifestyle-related factors.
Grazing’s frequency and associations with obesity, psychopathology, and loss of control eating in clinical and community contexts: A systematic review	Teodoro, M.C.; Conceição, E.M.; de Lourdes, M.; Alves, J.R.; Neufeld, C.B.	2021	Examine and critically analyze research focused on the prevalence of grazing behaviors in both clinical and community populations. The review also explores how grazing is associated with eating disorder psychopathology, loss of control eating, psychological distress, and variables linked to body weight.
The Impact of a Web-Based Mindfulness, Nutrition, and Physical Activity Platform on the Health Status of First-Year University Students: Protocol for a Randomized Controlled Trial	Trottier, C.F.; Lieffers, J.R.L.; Johnson, S.T.; Mota, J.F.; Gill, R.K.; Prado, C.M.	2021	Investigate how a web-based wellness platform influences perceived stress levels among first-year university students, while also examining its effectiveness in improving dietary quality.
Patterns of Physical Activity Among University Students and Their Perceptions About the Curricular Content Concerned With Health: Cross-sectional Study	Verma, A.K.; Singh, G.; Patwardhan, K.	2022	Assess the distribution of physical activity levels—ranging from inactive to highly active—among students at Banaras Hindu University, and explore their perspectives on how effectively the university curriculum informs and motivates them to maintain an active lifestyle.
Healthy Lifestyles of University Students in China and Influential Factors	Wang, D.; Xing, X.; Wu, X.	2013	Explore the lifestyle habits related to health among university students in China, identify the key determinants shaping these behaviors, and analyze how these findings could inform and improve future health promotion strategies.
The Relationship between Exercise and Psychotic Symptoms in College Students: A Cross-Sectional Analysis	Ye, Y.; Tang, H.	2023	Investigate the association between physical exercise and the occurrence of psychotic experiences among university students in the United States.
Circadian rhythms and sleep quality among undergraduate students in China: The mediating role of health-promoting lifestyle behaviours	Zhang, S.; Zhang, N.; Wang, S.; Hong, J.; Li, F.; Guo, H.; Lv, Z.; Wang, Y.; Wang, W.; Wu, W.	2023	Examine how circadian rhythm patterns relate to sleep quality among undergraduate students in China, with a focus on the mediating influence of lifestyle behaviors that support overall health.
Development and Validation of a Comprehensive Well-Being Scale for People in the University Environment (Pitt Wellness Scale) Using a Crowdsourcing Approach: Cross-Sectional Study	Zhou, L.; Parmanto, B.	2020	Develop and assess the validity of a detailed measurement tool aimed at evaluating well-being among individuals in university environments, encompassing students, academic personnel, and administrative staff.

Table reports all studies included in the scoping review and their respective objectives.
